# VIRO-TypeNed, systematic molecular surveillance of enteroviruses in the Netherlands between 2010 and 2014

**DOI:** 10.2807/1560-7917.ES.2016.21.39.30352

**Published:** 2016-09-29

**Authors:** Kimberley S M Benschop, Janette C Rahamat-Langendoen, Harrie G A M van der Avoort, Eric C J Claas, Suzan D Pas, Rob Schuurman, Jaco J Verweij, Katja C Wolthers, Hubert G M Niesters, Marion P G Koopmans, on behalf of VIRO-TypeNed

**Affiliations:** 1 Center for Infectious Disease Control, National Institute for Public Health and the Environment, Bilthoven, the Netherlands; 2 Department of Medical Microbiology, University Medical Center Groningen, Groningen, the Netherlands; 3 Current address: Department of Medical Microbiology, Radboud University Medical Center, Nijmegen, the Netherlands; 4 Department of Medical Microbiology, Leiden University Medical Center, Leiden, the Netherlands; 5 Department of Viroscience, Erasmus Medical Center, Rotterdam, the Netherlands; 6 Department of Virology, University Medical Center Utrecht, Utrecht, the Netherlands; 7 Laboratory of Medical Microbiology and Immunology, St Elisabeth Hospital, Tilburg, the Netherlands; 8 Department of Medical Microbiology, Academic Medical Center, Amsterdam, the Netherlands; 9 http://www.rivm.nl/Onderwerpen/T/Type_Ned/Type_Ned_Virologie

**Keywords:** VIRO-TypeNed, non-polio enterovirus, molecular surveillance, types, circulation patterns, immunity

## Abstract

VIRO-TypeNed is a collaborative molecular surveillance platform facilitated through a web-based database. Genetic data in combination with epidemiological, clinical and patient data are shared between clinical and public health laboratories, as part of the surveillance underpinning poliovirus eradication. We analysed the combination of data submitted from 2010 to 2014 to understand circulation patterns of non-polio enteroviruses (NPEV) of public health relevance. Two epidemiological patterns were observed based on VIRO-TypeNed data and classical surveillance data dating back to 1996: (i) endemic cyclic, characterised by predictable upsurges/outbreaks every two to four years, and (ii) epidemic, where rare virus types caused upsurges/outbreaks. Genetic analysis suggests continuous temporal displacement of virus lineages due to the accumulation of (silent) genetic changes. Non-synonymous changes in the antigenic B/C loop suggest antigenic diversification, which may affect population susceptibility. Infections were frequently detected at an age under three months and at an older, parenting age (25–49 years) pointing to a distinct role of immunity in the circulation patterns. Upsurges were detected in the summer and winter which can promote increased transmissibility underlying new (cyclic) upsurges and requires close monitoring. The combination of data provide a better understanding of NPEV circulation required to control and curtail upsurges and outbreaks.

## Introduction

Enteroviruses (EVs) are widespread viruses circulating globally. More than 100 types, classified to the four species A to D within the genus *Enterovirus* of the *Picornaviridae* family, are known to infect and cause disease in humans [1] The epidemiology of enteroviruses is characterised by the occurrence of seasonal peaks in the summer and temporal outbreaks that can be associated with life-threatening EV infections [2]. Clinical manifestations vary and range from asymptomatic or mild respiratory or gastrointestinal symptoms to severe and even fatal cases of myocarditis, neonatal sepsis, and central nervous system infections [3,4]. Severe and fatal cases often occur in children younger than five years or immunocompromised individuals [5-11]. Unfortunately, treatment options are limited and specific antivirals are not yet available [12,13]. Enteroviruses evolve by genetic diversification and recombination [14-17], which may affect their virulence [18-30]. In the Asian Pacific Region, EV-A71 (genotype C4) has been causing large outbreaks of hand, foot and mouth disease (HFMD) with severe complications since 2008 [22,23]. In a recent outbreak in the United States (US) and in Europe, an EV-D68 variant has been associated with outbreaks of severe respiratory disease and possibly paralysis [24-30]. While vaccination is a proven control strategy for some picornaviruses (poliovirus (PV), hepatitis A virus), little is known about the impact on population level of non-polio enteroviruses (NPEVs), even though these are among the most common endemic viruses. Knowledge on trends of NPEV illness comes from the decade-old EV surveillance programmes that have been implemented globally, where typing of enteroviruses from clinical samples from patients with polio-like illness is a cornerstone in the PV eradication campaign. Typing has historically been done by use of antigenic characterisation of virus isolates from routine diagnostic laboratories that used cell culture as the primary diagnostic method. Now laboratories are switching more and more to molecular detection and typing methods [31,32]. A major drawback of the widespread introduction of fast molecular diagnostic methods is that the surveillance spin-off of NPEV from the EV surveillance programme is no longer routinely available, and that molecular typing is dedicated to a few larger diagnostic and university hospital centres. To compensate for this, we have launched a collaborative molecular surveillance programme in 2010, in which sequence-based surveillance was introduced (VIRO-TypeNed) [33]. 

Here, we present an analysis of data submitted through this novel surveillance system from 2010 through 2014, which provides a better understanding of NPEV circulation in relation to seasonal epidemics and outbreaks.

## Methods

### Sampling and laboratory enterovirus diagnostic testing

Stool, respiratory, cerebrospinal fluid (CSF), blood and vesicle fluid samples from patients of different age groups, admitted to or visiting the hospital with an EV-associated illness, were sent to the clinical virology laboratories for testing. Symptoms varied from mild to severe respiratory illness, fever and appearance of vesicles to central nervous symptoms such as meningitis. Enterovirus testing were done by enterovirus-specific PCR tests [34,35], which are based on the conserved 5’UTR and enable detection of both PV and NPEVs, including those that do not grow in cell culture [36,37].

### Virus characterisation of 5’UTR-positive samples

Positive samples were characterised directly from clinical material or culture-positive samples by sequencing the VP1 gene [31,38-40]. The VP1 partial sequences obtained were used as input in the typing tool with an automated algorithm to assign the species and (sub)type of the sequences entered [41]. In addition, samples that could not be typed, in particular those with suspicion of PV infection, were sent to the reference laboratory (National Institute for Public Health and the Environment (RIVM)) for cultivation on the PV-specific cell line L20B to document the absence of wild-type PV circulation. Laboratories that do not perform typing of EVs are encouraged to send EV isolates or 5’UTR-positive samples to the RIVM for exclusion of PV and further characterisation.

### Reporting of data for EV surveillance by VIRO-TypeNed

The concept of VIRO-TypeNed has been described in detail elsewhere [33]. In short, participating Dutch clinical virology laboratories and the RIVM agreed on a consensus typing method described by Nix et al. [42] and on sharing of anonymised data in compliance with privacy rules via a secured web-based database. For each patient with a positive 5’UTR sample, at least one sequence of the VP1 gene generated by the Nix method is shared [40,42]. Sequences generated by other protocols can be included as well [43], but can only be included in the phylogenetic analysis when the region is compatible with the region generated with the Nix protocol. The VIRO-TypeNed platform includes a sequence-based typing tool with an automated algorithm to assign the species and (sub)type of the sequences entered, thus assuring comparability between the laboratories [41]. When available, a minimum set of clinical and epidemiological data are included with the submitted sequences, consisting of age, sex, date and type of sample, hospitalisation, travel history, clinical symptoms (skin, neurological, respiratory, enteric and other) and mortality.

### Data analysis

We analysed data submitted during the first five years of the VIRO-TypeNed project for trends, clusters and genetic diversity of common enteroviruses. Using data from the classical enterovirus surveillance containing data dating back to 1996 [32], the circulation patterns of the types were defined as endemic cyclic or as epidemic: (i) types with an endemic cyclic pattern of circulation were characterised by (predictable) seasonal increases every two to four years, with low detection levels in intervening years and (ii) types with an epidemic pattern were characterised by a unique outbreak in a given year while being rare (detection level n < 10) for at least 10 years before the given year [32].

To identify potential viral factors underlying the circulation patterns, all available partial VP1 gene sequences, which included the putative immunogenic B/C loop [44], were aligned and analysed for nucleotide and amino acid changes between lineages using the Simmonics sequence editor [45]. Pairwise distribution based on the nucleotide sequences of VP1 was calculated by MEGA6 [46] and used to set a demarcation cut-off to define lineages [47,48]. With the exception of EV-A71, classification of many NPEVs into lineages is not standardised. In cases where there is no uniform accepted lineage classification, lineages were designated alphabetically. EV-D68 lineages were designated as proposed by Tokarz et al. (A–C) [49] and Meijer et al. (I–III) [50].

## Results

In the period from 2010 through 2014, six diagnostic and university hospital laboratories from different parts of the Netherlands participated in the network (University Medical Center Groningen, Leiden University Medical Center, Erasmus Medical Center Rotterdam, University Medical Center Utrecht, St Elisabeth Hospital Tilburg and Academic Medical Center Amsterdam). The RIVM reported genotyping results of samples referred by 17 of 26 virology laboratories across the Netherlands that do not perform typing. A total of 1,917 EV-positive samples that had been genotyped were reported during the study period. The EV-B species was the most frequently detected species in all five years taken together, accounting for 70.3% (n = 1,347) of the viruses detected. The EV-A species comprised the second major species (24.4%, n = 467). The EV-C species and EV-D species (all identified as type EV-D68) accounted for 1.4% (n = 25) and 4% (n = 77), respectively, of the viruses detected. Oral PV vaccine (OPV) strains were detected in four patients; two cases in 2010 with OPV2 and OPV3, respectively, and two cases in 2011 also with OPV2 and OPV3, respectively. The five most commonly reported types and their ranking varied each year and accounted in total for 49–63% of the infections identified (Table).

**Table:** The five most frequent non-polio enterovirus infections collected by VIRO-TypeNed, the Netherlands, 2010–2014 (n = 1,917)

Ranking	2010	2011	2012	2013	2014
1	CV-A9	E-25	E-18	CV-B3	E-16
2	EV-A71	E-7	CV-A6	CV-A9	E-25
3	E-30	CV-B3	E-9	E-30	CV-A6
4	EV-D68	CV-B4	CV-A16	EV-A71	CV-A16
5	CV-A16	E-9	E-5	CV-A6	EV-D68

The ranking is based on the number of cases recorded.

**Table. The five most frequent non-polio enterovirus infections collected by VIRO-TypeNed, the Netherlands, 2010–2014 (n = 1,917)**

### Endemic circulation

**Figure 1:**
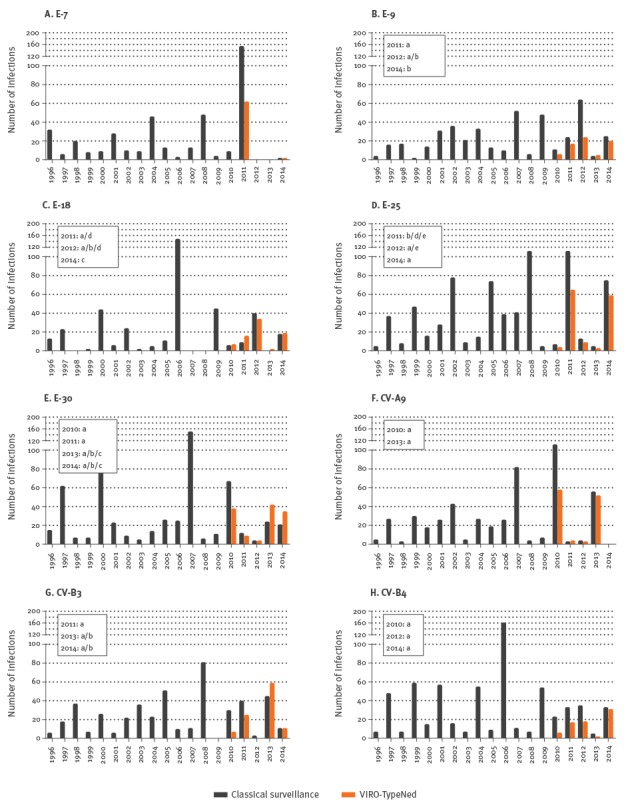
Distribution of endemic enterovirus types, the Netherlands, 1996–2014 (classical surveillance; n = 4,098) and 2010–2014 (VIRO-TypeNed; n = 714)

Types that were characterised as endemic cyclic were E-25 (n = 136), E-30 (n = 123), CV-A9 (n = 113), CV-B3 (n = 98), E-18 (n = 73), CV-B4 (n = 69), E-9 (n = 67) and E-7 (n = 62); they occurred with sharp peaks every two to four years (Figure 1). While there was no clear pattern indicative of evolution, phylogenetic clustering (data not shown) suggested the circulation of distinct genetic lineages that were defined by genetic changes in different motifs of the VP1 gene. E-9, E-18, E-25, E-30 and CV-B3 lineages were temporally defined (Figure 1), indicating continuous temporal displacement of variants. CV-A9 and CV-B4 showed no sequence divergence between the years (Figure 1). All E-7 strains were collected in 2011 and were genetically similar. For all types, we found that the genetic changes were primarily silent. In addition to these silent changes, E-25, CV-B3 and E-9 variants showed amino acid substitutions within the B/C loop, suggesting antigenic diversification over time. E-25 strains encoded a valine (V, lineage b and d) or threonine (T, lineages a and e) at position 78 and an aspartic acid (D, lineage b and a) or asparagine (N, lineage d and e) at position 86 of the E-25 VP1 protein (amino acid numbering based on GenBank accession number HM03119). CV-B3 strains encoded a lysine (K, lineage a) or asparagine (N, lineage b) at position 85 of the CV-B3-VP1 protein (numbering based on GenBank accession number JX312064) and E-9 carried an asparagine (N, lineage a) or aspartic acid (D, lineage b) at position 84 of the E-9 VP1 protein (numbering based on GenBank accession number AF524866) [44,51,52].

Lineages are indicated in boxes in the graphs. Black bars indicate classical surveillance. Orange bars indicate VIRO-TypeNed data.

### Epidemic circulation

**Figure 2:**
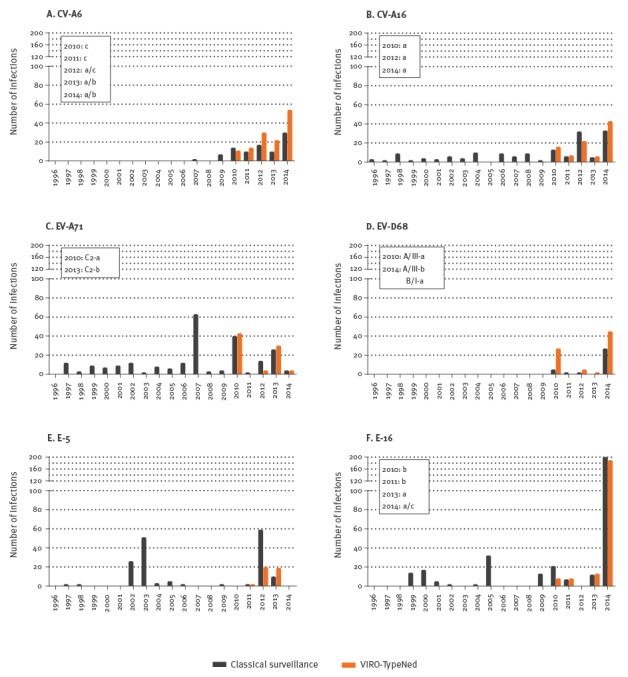
Distribution of epidemic enterovirus types, the Netherlands, 1996–2014 (classical surveillance; n = 938 and 2010–2014 (VIRO-TypeNed; n = 619)

Types that were characterised as epidemic were E-16 (n = 212), CV-A6 (n = 126), CV-A16 (n = 89), EV-A71 (n = 77, of which 69 were C2), EV-D68 (n = 77) and E-5 (n = 38). E-16 was frequently reported in 2014 and was detected in 29% of the infections in the summer of 2014, which was twice as much than any other type detected in previous years (Figure 2).

Lineages are indicated in boxes in the graphs. Black bars indicate classical surveillance. Orange bars indicate VIRO-TypeNed data.

Of interest is that CV-A6, CV-A16 and EV-A71 has continuously been detected in an endemic cyclic pattern since 2010 (Figure 2). Sequence divergence between CV-A6 lineages and EV-A71 C2 sublineages were primarily silent and were temporally defined (Figure 2), indicating the same continuous displacement of variants as seen for the endemic types. In contrast, CV-A16 variants did not display any significant sequence variations defining temporal clustering (Figure 2).

The shift from epidemic to endemic cycle was observed also for EV-D68. An upsurge of EV-D68 has been observed in 2010, after very low frequency for 14 years, and again in 2014 (Figure 2). The EV-D68 strains from 2010 and 2014 clustered as temporally defined sublineages within the previously assigned lineages A/III and B/I (Figure 2). Sequence divergence between the two lineages showed amino acid substitutions within the B/C loop; the variants of the 2014 B/I lineage were found to encode D90, T92 and alanine (A)95 of the VP1 protein (amino acid numbering based on GenBank accession AB061487). The variants of the 2010 and 2014 A/III lineages were found to encode N90, A92 (2010, lineage a) or T92 (2014, lineage b), and glutamic acid (E)95 (Figure 2).

### Patient characteristics

Using additional patient data submitted to VIRO-TypeNed, we analysed factors such as age of infection, sex and clinical symptoms and their influence on the occurrence of endemic and epidemic patterns of the different types. The female:male sex ratio varied considerably between the types and there was no difference between endemic and epidemic types. Infections were frequently or equally found among girls for the endemic types CV-A9 (ratio: 0.9) and CV-B4 (ratio: 0.9), and the epidemic types EV-D68 (ratio: 0.7) and CV-A6 (ratio: 1). For most types, infections were frequently found among boys for the endemic types E-30 (ratio: 1.1), CV-B3 (ratio: 1.2), E-25 (ratio: 1.3), E-5 (ratio: 1.9), E-18 (ratio: 1.7), E-9 (ratio: 1.5), E-18 (ratio: 1.7) and E-7 (ratio: 2.4), and the epidemic types E-16 (ratio: 1.1), CV-A16 (ratio: 1.1) and EV-A71 (ratio: 1.5). 

Overall, infections were detected in cases of all ages (< 28 days to 85 years) with a majority of cases younger than five years (n = 1,067/1,249; 56%). Cases infected with the epidemic types E-5 and E-16 were predominantly younger than three months (n = 18/38; 47% and n = 159/212; 75%, respectively). In contrast to the EV-B types characterised as either endemic or epidemic, cases infected with EV-A types were predominantly one to two years of age (25–35%): CV-A6 (n = 38/126), CV-A16 (n = 24/89), EV-A71 (n = 19/77) and the EV-D type EV-D68 (n = 25/77). A number of viruses were also frequently found in adults (at parenting age, 25–45 years) in 3–20% of the cases: E-30 (n=15/123), CV-A9 (n=11/113), E-18 (n = 8/73), CV-A6 (n = 14/126), CV-A16 (n = 5/89) and EV-A71 (n = 3/77). Adult infections with EV-D68 were also frequently found among cases aged 45 to 65 years (n = 12; 16%). No adults with E-25, CV-B3 and E-9 infections were identified. Adults infected with other EV-B comprised less than 1% of the infections. Clinical information was reported for only 5–30% of the cases. Nonetheless, it was found that all HFMD disease reports were attributed to EV-A infections (10/25, p = 0.003). Neurological symptoms were statistically more frequently reported among EV-B-infected cases (85/129) compared with only a few EV-A-infected cases (8/21) (p = 0.027).

### Seasonal distribution

Because typing data were linked to date of isolation, VIRO-TypeNed enabled direct analysis of seasonal trends of new upsurges of the common types as well as trends between the types. A clear seasonal distribution was observed in the years 2010, 2013 and 2014, with 15–29% of the EV infections observed in July and August of those years. In some years, there were clear seasonal peaks in winter, contrary to the summer peaks that are considered typical for enteroviruses. In 2011 and 2012, most EV infections detected in those years were found in the late fall and winter 2011/12 (October to February; detection ranged from 16% in October 2011 to 9% in February 2012). In contrast, not many summer infections were reported in those years (11% in July 2011 and 6% in August 2012). When an upsurge was detected after more than 10 years of low EV activity, the epidemic types CV-A6, CV-A16, EV-D68 and E-5 were predominantly detected in the winter. For CV-A6, CV-A16 and EV-D68, the seasonality shifted towards summer and fall in the following years.

## Discussion 

In this paper, we describe the systematic surveillance of NPEVs for the Netherlands through the VIRO-TypeNed system, which is based on molecular typing of pathogens.

By analysing virus sequence data in combination with epidemiological and patient data, we show for the first time in a standardised manner the circulation patterns of NPEVs in the Netherlands enabling a better understanding of NPEV circulation, which is required to control and curtail outbreaks and upsurges. With the knowledge on the endemic cyclic patterns, a rise in the number of positive cases two to four years after the last upsurge warrants vigilance because it could indicate an imminent upsurge/outbreak [53]. Of interest is the shift from an epidemic to an endemic pattern for CV-A6, CV-A16, EV-A71 and EV-D68; this should be taken into account when monitoring rare types. For the EV-A viruses, the observed shift could be a surveillance artefact due to the change in detection methods from culture to molecular, as molecular methods have increased sensitivity and capture viruses that are more difficult to culture, such as these EV-A viruses [31-33,36]. Another explanation could be a change in pathogenicity. We found a low level of circulation of EV-D68 (before 2010) and EV-C viruses, which is consistent with other studies (reviewed by [31]) and is suggested to be related to a low pathogenicity of these types [43]. Indeed, in 2010 and 2014, hospitals in Europe and the US reported increased detection of EV-D68 in respiratory samples from cases with severe respiratory disease [24-29,50,54-57]. 

The increase was related to genetic changes that could have driven a more severe pathogenicity rather than to changes in detection methods [50]. Unfortunately, clinical data were missing in the majority of cases of other viruses and more data are required to investigate whether pathogen drift could have additionally contributed to the increased detection of other NPEVS in certain years.

The data further suggest that the epidemic/endemic cyclic patterns might be driven by immunity; this could be due to antigenic diversification, waning immunity or simply lack of immunity. The frequency of infections at an extremely young age and at parenting age [58-60] suggests lack of protection by maternal antibodies [61]. Lack of immunity or waning immunity can be inversely related to the endemic cyclic patterns of the different types/strains; the types/strains that adults are exposed to are different from those circulating during their childhood when they frequently came in contact with EVs. An adult’s immunity profile is thus directed against EV types not currently circulating, which has led to a high proportion of susceptible adults. In the case of antigenic diversification, the immunity that was predominantly built up in previous years may be lacking (loss of neutralisation capacity) or not effective against currently circulating antigenic variants (altered neutralisation capacity) [62]; this also leads to a high proportion of susceptible adults [24,50,54]. For EV-D68, the high proportion of infection among adult cases aged 45 to 65 years, and the divergence between the two lineages showing amino acid substitutions within the B/C loop, indeed suggest that antigenic diversification leading to altered neutralisation capacity plays a role in adult infections [50,54]. In contrast, E-30, CV-A9, E-18, CV-A6, CV-A16 and EV-A71, all frequently observed in adult infections, showed no antigenic diversification. However, we cannot rule out the occurrence of antigenic diversification among these types, as antigenic epitopes can also be found among other exposed VP1 loops and on other capsid proteins [44,51,52] not characterised by the Nix protocol.

Furthermore, no adults were identified among E-25, CV-B3 and E-9, strains proposed to have antigenic diversification. This would suggest that other factors act as a transmission bottleneck, such as differential receptor expression between adults and children. It has been suggested that viral characteristics such as receptor usage can account for the differential age of infection with several EV types and HPeV [19,63].

Another factor affecting the circulation patterns is seasonality. It has been suggested that infection frequency is dependent on the number of contacts or transmissions, which can be influenced by the season or the weather. The frequency of contacts is highest during the winter months [64-66], which could spur widespread transmission after an initial introduction and lead to unexplained illness outbreaks during winter season. The observed winter peak could be related to the introduction of fast molecular methods that allowed rapid screening of samples throughout the years, revealing a more diverse seasonal pattern. EV should therefore also be considered in the differential diagnosis during winter seasons, contrary to the dogma describing EV infections as seasonal summer infections.

To investigate and understand the role of these factors (antigenic diversification, lack of or waning immunity, and seasonality) on the circulation pattern, full-length genetic and phenotypic analysis in combination with sero-population studies need to be conducted over an extended period. VIRO-TypeNed provides a platform to analyse these data in relation to one another.

Knowledge about the endemic/epidemic patterns can be used to investigate the possibility of type-specific vaccines [67]. Meanwhile, the data can be used to pre-screen intravenous immunoglobulin (IVIG) products, where the knowledge of which types are currently circulating can enable a more effective use of IVIGs.

Furthermore, with the current developments in the antiviral field, EV infections may soon be classified as treatable rather than life-threatening [13]. The drugs currently in development show type-specific efficacy [68,69] and use requires the knowledge of which types are currently circulating or may cause an outbreak.

While VIRO-TypeNed provides data on NPEVs, the system also allows reporting the detection of PV. PV circulation might occur through inadvertent introduction of OPV, vaccine-derived PV (VDPV) or even wild-type PV, or via faecal excretions from migrants or travellers returning from endemic or OPV-using countries. Any type of PV isolation in the Netherlands leads to a public health alert because there is a large unvaccinated group (3% of the population) refusing vaccination for religious reasons that live in a closely isolated community (the Bible Belt). The 5’UTR PCR is able to detect all EVs including PV. Over the period studied, four OPV strains were reported. They had been detected by direct genotyping from clinical samples and had already been notified to the RIVM, and preventive actions for further spread had been taken. Given the very low circulation rate of PV in the Netherlands in non-epidemic years, EV-positive samples from which unique NPEV sequences are generated are considered PV-negative. However, as positive untyped samples can contain PV, laboratories are encouraged to send these samples, in particular those with suspicion of PV infection, to the RIVM for cultivation on L20B cells. As such, the surveillance capacity to exclude PV circulation in a molecular era is maintained.
